# Occurrence of Aflatoxin M1 in Raw Milk from Manufacturers of Infant Milk Powder in China

**DOI:** 10.3390/ijerph15050879

**Published:** 2018-04-28

**Authors:** Songli Li, Li Min, Gang Wang, Dagang Li, Nan Zheng, Jiaqi Wang

**Affiliations:** 1 Laboratory of Quality & Safety Risk Assessment for Dairy Products of Ministry of Agriculture (Beijing), Institute of Animal Science, Chinese Academy of Agricultural Sciences, Beijing 100193, China; lisongli@caas.cn (S.L.); jiaqiwang@vip.163.com (J.W.); 2 State Key Laboratory of Livestock and Poultry Breeding, Institute of Animal Science, Guangdong Academy of Agricultural Sciences, Guangzhou 510640, China; min1988317@163.com (L.M.); wanggang@gdaas.cn (G.W.); lidagang@gdaas.cn (D.L.); 3 Milk and Dairy Production Inspection Center of Ministry of Agriculture (Beijing), Beijing 100193, China

**Keywords:** aflatoxin M1, raw milk, infant milk, season, China

## Abstract

This survey was performed to investigate the occurrence of aflatoxin M1 (AFM1) contamination of raw milk from manufacturers of infant milk powder in China. A total of 1207 raw milk samples were collected overall from four seasons of 2016 in Northeast China, Northwest China, Northern China, and Central China (11 provinces and one municipality). Results showed that 56 of the 1207 raw milk samples (4.64%) were positive for AFM1, which were obtained from Heilongjiang (two samples), Gansu (one sample), Shaanxi (46 samples), Beijing (one sample), and Hunan (six samples) provinces. None of the raw milk samples from manufacturers of infant milk powder exceeded the Chinese limit (62.5 ng/L) in 2016. Only a few raw milk samples were not suitable for use in infant milk according to EU (European Union) or U.S. infant milk limits. Furthermore, based on this survey and previous studies, it is particularly important to avoid AFM1 contamination in raw milk during the winter.

## 1. Introduction

Milk is considered to be a perfect natural food for all age groups of consumers due to its high nutritional value [1]. Therefore it can be used to manufacture functional dairy foods [2]. The growing demand of human consumption for functional dairy foods has driven the dairy market in the world [3]. However, it has the greatest potential risk for introducing aflatoxin M1 (AFM1) into the human diet [4]. AFM1 is classified as a Group 1 toxin [5], causing immunosuppression, carcinogenicity and teratogenesis [6]. The risk management in order to avoid the AFM1 contamination in raw milk is strongly related with the control of AFB1 (aflatoxin B1) intake by dairy animals. A recently study reported that occurrence rates of AFB1 were more than 83.3% in feeds in China [7]. Obviously, AFM1 contamination of raw milk is a substantial public health concern [8]. Furthermore, AFM1 is heat stable, it is only degraded at temperatures of at least 250 °C. If raw milk is contaminated by AFM1, the residues of AFM1 remain stable in the final milk products even after heat processing, such as milk powder [9].

The occurrence of AFM1 in commercially available milk products has led to an impetus to establish measures to control AFM1 contamination, especially in those designed for infants [4]. The exposure of infants to AFM1 is a major concern because of their high milk intake [10]. The capacity for biotransformation of toxins in infants is generally slower than that in adults, which may result in a longer circulation time of the toxin, and then neonatal growth retardation [11]. Obviously, infants are the most susceptible population to the deleterious effects of AFM1 [12]. Most countries have set maximum permissible levels for AFM1 in raw milk and milk products. These range from 50 ng/L in the European Union (EU) to 500 ng/L in the United States (U.S.) [13]. However, because of the susceptibility of infants to AFM1, both the EU and U.S. prescribe a limit of 25 ng/L AFM1 for infant milk [11].

In September 2008, high concentrations of melamine were detected in infant milk powder, which led to severe sickness in infants in China [14]. Since then, the Chinese government has paid more attention to control the safety of raw milk and infant milk powder. The overall management systems were used in dairy farms in the different regions of China, such as carrying out national inspections on milk safety, funding dairy farmers to improve breeding conditions and training dairy farms with safety skills [15]. The maximum level of AFM1 permitted is 62.5 ng/L for infant milk in China (based on GB2761-2017, Chinese National Standards). However, there are very few data to show the frequency with which AFM1 occurs in raw milk used to produce infant milk powder in China. To address this problem, 1207 raw milk samples were collected from manufacturers of infant milk powder in 2016, in order to better characterize the safety of infant milk powder in China.

## 2. Material and Methods

### 2.1. Collection of Samples

To analyze the representative samples of raw milk from manufacturers of infant milk powder in China, samples were collected by a convenience sampling method, as previously described [16]. A total of 1207 raw milk samples were collected over all four seasons (480 in spring, 229 in summer, 128 in autumn, and 370 in winter) during 2016 in four different areas (Northeast China, Northwest China, Northern China, and Central China).

Of these, 670 samples were collected from Northeast China (from Heilongjiang, Jilin, and Liaoning provinces), 409 samples were collected from Northwest China (from Gansu, Ningxia, and Shaanxi provinces), 64 samples were collected from Northern China (from Beijing, Hebei, and Henan provinces), and 64 samples were collected from Central China (from Anhui, Hubei, and Hunan provinces).

### 2.2. Sample Preparation

The raw milk samples were stored at 4 °C until analysis. Before analysis, liquid samples were centrifuged at 3000 *g* for 10 min at 4 °C, the upper cream layer was completely removed, and then the supernatants were collected for analysis. All analyses were completed before the expiration date of the samples.

### 2.3. Analysis of AFM1 by ELISA

Quantitative analysis of AFM1 in skimmed milk samples was carried out using an enzyme-linked immunosorbent assay (ELISA) test kit (RIDASCREEN Aflatoxin M1, R-Biopharm AG, Darmstadt, Germany). The test was used according to the manufacturer’s instructions. 

Concentrations of AFM1 were calculated from calibration curves that were obtained using AFM1 standards (0, 5, 10, 20, 40, and 80 ng/L) in the test kit. A sample was considered to be positive for AFM1 if the levels exceeded the minimum detection limit for the assay (5 ng/L). A sample with AFM1 concentration greater than 80 ng/L was diluted with sample diluent solution from the test kit and reanalyzed.

In the present study, the validation variables were calculated and shown as follows: limit of detection (LOD) = 5 ng/L, limit of quantification (LOQ) = 8.5 ng/L, recovery = 85–110%, and relative standard deviation calculated under repeatability conditions (RSDr) < 8%. The results of validation variables suggested that the data in this study were acceptable for further analysis.

### 2.4. Statistical Analysis

All raw milk samples were analyzed in duplicate. AFM1 concentrations were expressed as mean ± Standard deviation (SD) in order to show the occurrence of AFM1 in raw milk from manufacturers of infant milk powder in four regions of China (Northeast China, Northwest China, Northern China, and Central China) during 2016. The AFM1 concentrations were statistically analyzed by using the nonparametric test, followed by Mann-Whitney comparisons, using SPSS Statistics17.0 (SPSS, Inc., Chicago, IL, USA).

## 3. Results and Discussion

In total, 56 of the 1207 raw milk samples (4.64%) were positive for AFM1 (>5 ng/L). Of these, only two samples were positive in Northeast China, 47 samples were positive in Northwest China, one sample was positive in Northern China, and six samples were positive in Central China (Table 1). The highest concentration of AFM1 (60 ng/L) was found in Central China (in a sample from Hunan province). There was no significant difference in the concentrations of AFM1 in raw milk from manufacturers of infant milk powder in four regions of China (*p* > 0.05). All the concentrations were below the maximum permissible level for AFM1 in raw milk and milk products in China and in U.S. (500 ng/L). Recently, Li et al. [17] reported the level of AFM1 contamination of raw milk in China. The results of this comprehensive survey showed that an AFM1 contamination level of 1.1% of raw milk samples exceeded the EU limit (50 ng/L), but none exceeded the Chinese and U.S. limit (500 ng/L) in 2016. A series of measures that were introduced to control and improve raw milk and dairy processing are likely to have improved the safety of milk in China in recent years [18]. It is worth mentioning that the application of assurance quality systems in dairy industry (included the good manufacturing practices (GMPs), sanitation standard operating procedures (SSOPs), and hazard analysis critical control point (HACCP)) significantly improved the safety of milk [19,20]. Overall, the increasing awareness for establishing continuous monitoring systems were conducted for the aflatoxin issue in milk products worldwide [21].

**Figure 1:**
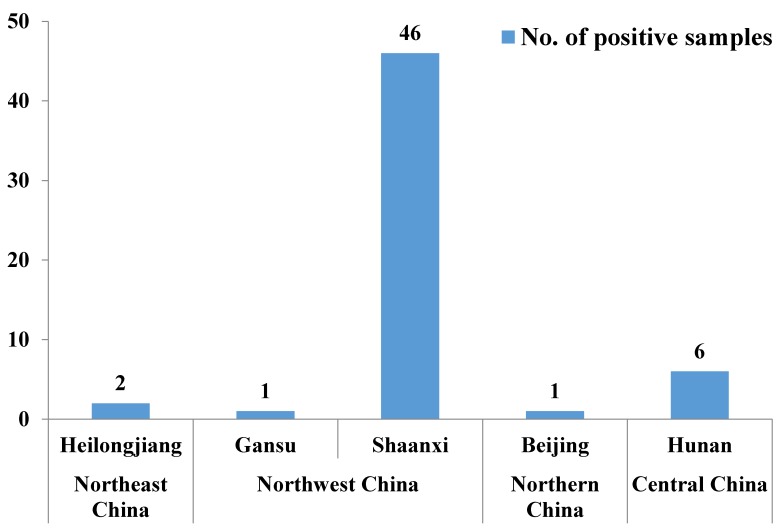
Number of AFM1-positive raw milk samples from manufacturers of infant milk powder in regions of China. 670 samples were collected from Northeast China (from Heilongjiang, Jilin, and Liaoning provinces), 409 from Northwest China (from Gansu, Ningxia, and Shaanxi provinces), 64 from Northern China (from Beijing, Hebei, and Henan provinces), and 64 from Central China (from Anhui, Hubei, and Hunan provinces).

As shown in Figure 1, the positive raw milk samples were obtained from Heilongjiang (two samples), Gansu (one sample), Shaanxi (46 samples), Beijing (one sample), and Hunan (six samples) provinces. The variations in AFM1-positive raw milk samples from different regions may be related to geographic and climatic differences as well as differences in feeding systems, farm management practices, and hygiene conditions [13]. It is important to identify the risk factors which cause the positive identification of AFM1 in raw milk. Michlig et al. [22] reported the risk factors associated with the presence of AFM1 in raw milk. Farm breeding intensification and the supplementation with commercial feed, maize, and cotton seed seem to be the risk factors that impact on the AFM1 milk contamination. It is therefore particularly important to monitor aflatoxin contamination in feed and AFM1 contamination in raw milk from manufacturers of infant milk powder in these areas. A study in India reported that the contamination level of AFM1 in infant formulas ranged from 143 to 770 ng/L [23], which would not be suitable for use in infant milk for export. Sixty-three infant formulas were randomly collected from pharmacies and supermarkets in Turkey, the contamination levels of AFM1 were 60–320 ng/L [24], which would be not suitable for use in infant milk for export. Forty eight out of 100 raw milk samples contained AFM1 (with an average of 26 ng/L), which were mainly used for the preparation of infant formulas, yogurts and other dairy products in South Korea [25]. A survey on the presence of AFM1 in the 14 leading brands of infant formulas marketed in Italy was conducted. AFM1 was found in two of 185 samples, but at levels below the EU legislation limit of 25 ng/L [26]. The occurrence of AFM1 in infant formulas from Spain was detected in 37.7% of the 69 samples, with a range of 0.6–11.6 ng/L [27]. Based on the results shown in Table 1, it should be noted that 13 raw milk samples (1.08%) would not have been suitable for use in infant milk in the EU and U.S. (maximum limit, 25 ng/L AFM1). None of raw milk from manufacturers of infant milk powder exceeded the Chinese limit (62.5 ng/L) in 2016.

**Figure 2:**
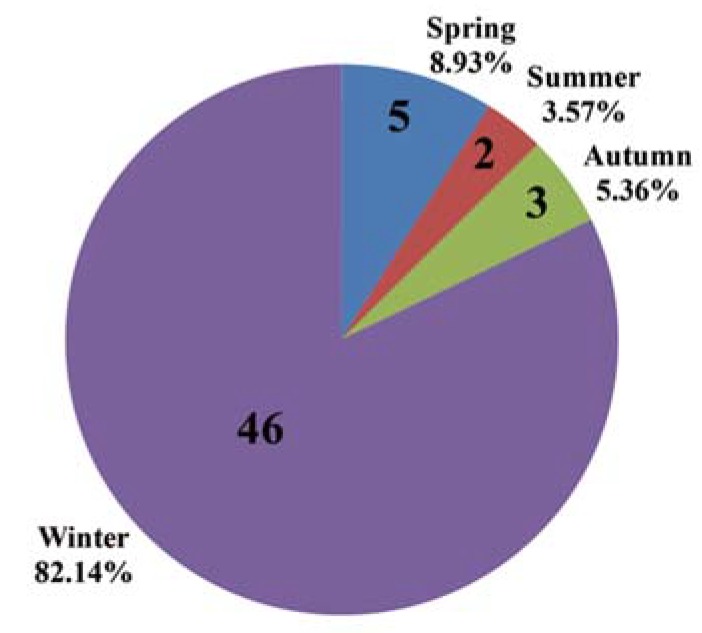
Distribution of AFM1-positive samples from manufacturers of infant milk powder in China over all four seasons. The numbers of positive samples collected were 5, 2, 3, and 46 in spring, summer, autumn, and winter, respectively.

A previous study showed that the prevalence of AFM1 contamination in raw milk samples in China was much higher in winter than in other seasons [17]. To obtain a systematic and unbiased understanding of the effect of season on AFM1 contamination of raw milk, the distribution of positive samples during all four seasons was assessed and is shown in Figure 2. The numbers of positive samples were 5, 2, 3, and 46 in spring, summer, autumn, and winter, respectively. Thus, it is clear that raw milk samples have a higher risk of AFM1 contamination in winter (82.14% of the total 56 positive samples). This seasonal trend in China is consistent with those in a wide range of other countries, including Croatia, Iran, Pakistan, Serbia, Thailand, and Turkey [4,28,29,30,31,32]. These studies confirmed that the occurrence of AFM1 in raw milk was more common in winter worldwide. The reason for this relates to lower availability of fresh green feed in winter, with the risk of aflatoxin contamination of silage and other stored feedstuffs being higher. High levels of AFM1 contamination in raw milk occur when cows are fed with highly contaminated feedstuffs [33]. It can be concluded that a low occurrence of AFM1 concentrations is the consequence of the systematic control of aflatoxin B1 in primary production of feed for dairy cows [34]. Thus, the adoption of good harvesting practices and the implementation of strict regulations should be pursued to reduce and avoid AFM1 contamination in winter [35].

## 4. Conclusions

A series of surveys have been performed to monitor the frequency of AFM1 contamination in raw milk and milk products in China during recent years [15,17,18,36,37,38]. It appears that the quality and safety of milk in China has improved in recent years, as indicated by the decrease in AFM1 contamination of milk samples. None of raw milk from manufacturers of infant milk powder exceeded the Chinese limit (62.5 ng/L) in 2016. Only a few raw milk samples were not suitable for use in infant milk according to EU or U.S. infant milk limits. In addition, raw milk samples have a higher risk of AFM1 contamination in winter. Therefore, good harvesting practices and strict regulations should be adopted during the winter season in China. 

## Figures and Tables

**Table 1:** Occurrence of aflatoxin M1 (AFM1) in raw milk from manufacturers of infant milk powder in four regions of China during 2016.

Area	Provinces	Positive/Total Samples (%)	Distribution of AFM1 Concentration (ng/L) in Positive Samples				Maximum (ng/L)	Mean ± SD (ng/L) ^e^
			<25 ^a^	25–50 ^b^	50–62.5 ^c^	62.5–500 ^d^		
Northeast China	Heilongjiang, Jilin, and Liaoning provinces	2/670 (0.30%)	1	1	0	0	25.5	20.7 ± 6.8
Northwest China	Gansu, Ningxia, and Shaanxi provinces	47/409 (11.49%)	42	4	1	0	55	19.9 ± 21.6
Northern China	Beijing, Hebei, and Henan provinces	1/64 (1.56%)	0	1	0	0	40	40
Central China	Anhui, Hubei, and Hunan provinces	6/64 (9.38%)	0	4	2	0	60	46.7 ± 10.4
Total	11 provinces + 1 municipality	56/1207 (4.64%)	43	10	3	0	60	14.8 ± 15.9

^a^ The maximum level of AFM1 permitted is 25 ng/L for infant milk in the EU and the U.S. ^b^ The maximum level of AFM1 permitted is 50 ng/L for milk and milk products in the EU. ^c^ The maximum level of AFM1 permitted is 62.5 ng/L for infant milk in China. ^d^ The maximum level of AFM1 permitted is 500 ng/L for milk and milk products in China and the U.S. ^e^ Mean ± SD is calculated from the value of the positive samples. There was no significant difference in the concentrations of AFM1 in raw milk from manufacturers of infant milk powder in four regions of China (*p* > 0.05).
